# Platycoside O, a New Triterpenoid Saponin from the Roots of *Platycodon grandiflorum*

**DOI:** 10.3390/molecules16064371

**Published:** 2011-05-26

**Authors:** Wen-Wei Fu, Jin-Nan Fu, Wen-Meng Zhang, Li-Xin Sun, Yue-Hu Pei, Ping Liu

**Affiliations:** 1 Institute of Liver Diseases, Shanghai Shuguang Hospital, Shanghai 201203, China; 2 Shanghai Clinical Key Laboratory of Traditional Chinese Medicine, Shanghai Shuguang Hospital, Shanghai 201203, China; 3 Key Laboratory of Liver and Kidney Diseases, Shanghai University of Traditional Chinese Medicine, Shanghai 201203, China; 4 E-Institute of Traditional Chinese Medicine Internal Medicine in Shanghai University, Shanghai University of Traditional Chinese Medicine, Shanghai 201203, China; 5 Institute of Microbiology, Jiangxi Academy of Sciences, Nanchang 330029, China; 6 School of Pharmacy, Shenyang Pharmaceutical University, Shenyang 110016, China

**Keywords:** platycoside O, *Platycodon grandiflorum*, triterpenoid saponin

## Abstract

A new unusual minor triterpenoid saponin, platycoside O (**1**), was isolated from the 75% EtOH extract obtained from the roots of *Platycodon grandiflorum*, together with four known saponins: platycoside M-3 (**2**), platycoside J (**3**), platycoside F (**4**) and platycoside B (**5**). The structure of **1** was determined as 3-*O*-β-D-glucopyranosyl-(1→6)-β-D-glucopyranosyl-2β,3β,16α,23-tetrahydroxyolean-12-en-24-methoxyl, 24-oxo-28-oic acid 28-O-β-D-xylopyranosyl-(1→4)-α-L-rhamnopyranosyl-(1→2)-α-L-arabinopyranoside on the basis of spectral analysis and chemical evidence.

## 1. Introduction

*Platycodon grandiflorum* (Campanulaceae) is a species of perennial flowering plant of the family Campanulaceae and the only member of the genus Platycodon. In traditional Oriental medicine, its root (*Radix Platycodi*) has been extensively used since ancient times as a traditional drug to treat coughs, colds, upper respiratory tract infections, sore throats, tonsillitis, and chest congestion [1]. In the northeastern of China and Korea, the root is also a popular ingredient in salads and traditional cuisine. Chemical investigation of P. radix revealed that triterpenoid saponins were the main chemical components [1], which exhibit a variety of pharmacological activities, such as anti-inflammatory [2,3], protective effects on the hepatotoxicity induced by chemicals [4,5], anti-antioxidant [6], anti-cancer [7,8] and adjuvant against hepatitis B antigen [9,10]. Till date, more than 40 triterpenoid saponins have been isolated from the roots of the plant [1,11,12,13,14,15,16,17,18,19,20,21]. 

In our previous papers, we reported the isolation and structural elucidation of eleven new triterpenoid saponins, including several unusual A-ring lactone triterpenoid saponins, from the roots of *P. grandiﬂorum* A. DC [13,14,15,16]. Further investigation on the polar fractions of *P. grandiﬂorum* led to the isolation from the EtOH extract obtained from the roots of a new unusual minor triterpenoid saponin based on the sapogenin platycogenic acid A, named platycoside O, together with four known compounds. To the best of our knowledge, the triterpenoid saponins based on the sapogenin platycogenic acid A had been isolated only in the plant and reported in a few publications [19,22].

## 2. Results and Discussion

**Figure 1:**
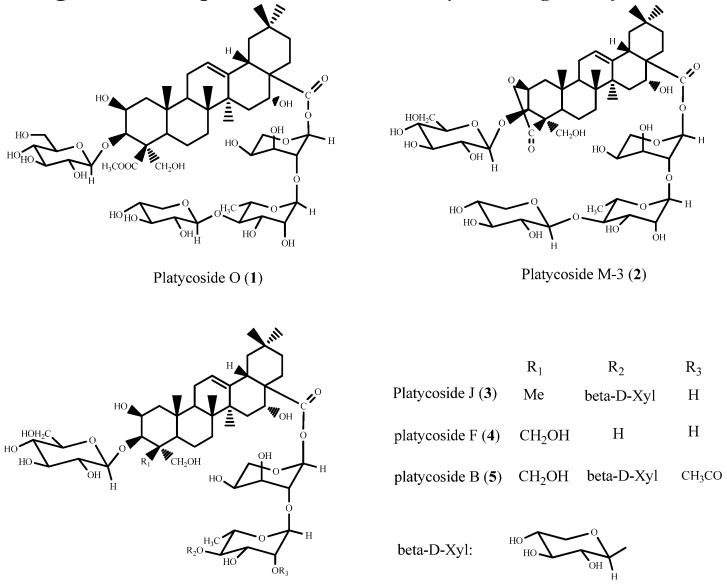
Compounds **1–5** from *Platycodon grandiﬂorum*.

The 75% EtOH extract from the roots of *Platycodon grandiﬂorum* were partitioned with aqueous EtOAc. The aqueous layer was separated by a macroreticular resin column to give the 60% EtOH eluates that upon drying afforded the total saponins. The total saponins were chromatographed on silica gel, a reverse-phase column, and ﬁnally on HPLC to afford the compound **1** and four known compounds (Figure 1).

Platycoside O (**1**) was a white amorphous powder, and its molecular formula was assigned to be C_53_H_84_O_25_ based on the high-resolution (HR)-FAB-MS spectrum. The spectral features and physicochemical properties revealed **1** to be a triterpenoid saponin. The IR spectrum exhibited absorptions at 3,421 cm^−1^ (OH), 1,738 cm^−1^ (ester carbonyl), 1,633 cm^−1^ (trisubstituted double bond), and 1,384 cm^−1^ (gem-dimethyl). Five tertiary methyl groups (δ 0.99, 1.14 × 2, 1.33, 1.55, 1.76) and one oleﬁnic proton (δ 5.63, br s) were observed in the ^1^H-NMR spectrum. The ^13^C-NMR spectrum showed five sp^3^ carbons at δ 17.6, 17.8, 24.8, 27.2, and 33.3, two sp^2^ oleﬁnic carbons at δ 123.1 and 144.4, four oxygenated methylene and methine carbons at δ 63.7, 70.2, 74.0, and 85.1 (Table 1), two carbonyl carbons at δ 170.5, and 175.9. The information on the ^1^H-NMR spectrum coupled with the ^13^C-NMR spectrum indicated that **1** has an 2β,3β,16α,23-trihydroxyolean-12-en-24,28-dioic acid skeleton [22]. The HMBC spectrum showed that correlation between H (δ_H_ 3.69) of a methoxy group (δ_H_ 3.69, δ_C_ 52.1) and the carbon (δ_C_ 170.5) at the C-24 carboxy group. A 2D NMR experiments such as COSY, DEPT, HMQC and HMBC and by the comparison with the data in the literature revealed that the aglycon was 2β,3β,16α,23-trihydroxyolean-12-en-24-methoxyl, 24-oxo-28-oic acid (24-methyl platycogenic acid A). 

The chemical shifts of C-3 (δ 85.1) and C-28 (δ 175.9) revealed that **1** was a bisdesmosidic glycoside. The ^1^H- and ^13^C-NMR spectra of **1** exhibited four sugar anomeric protons at δ 5.18 (d, *J* = 8.0 Hz), 5.27 (d, *J* = 8.0 Hz), 5.78 (br s), and 6.47 (d, *J* = 2.5 Hz) and carbons at δ 93.6, 101.2, 106.4, and 106.8 (Table 1). In the ^1^H-NMR spectrum, one doublet methyl signal at δ 1.74 (*J* = 5.5 Hz) belonging to rhamnose was observed. Acid hydrolysis of **1** gave a 1:1:1:1 ratio of arabinose, rhamnose, xylose, and glucose, which were analyzed by gas chromatography as their alditol acetates. The absolute conﬁgurations of sugars were shown to be L-arabinose, L-rhamnose, D-xylose, and D-glucose according to the method reported by Hara and coworkers [14,15]. 

**Figure 2:**
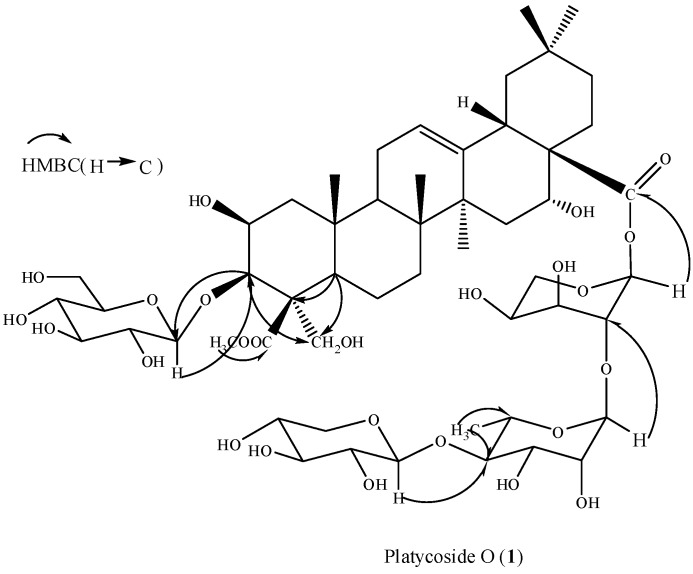
The structure and selected HMBC correlations of **1**.

All the monosaccharides of **1** were in pyranose forms, as determined by their ^1^H- and ^13^C-NMR spectral data as well as 2D NMR experiments. The β-anomeric conﬁgurations of the D-glucose and D-xylose units were determined by their *^3^J_H1,H2_* coupling constants (7.0–8.0 Hz). The α-anomeric conﬁguration of the L-arabinose was determined by its *^3^J_H1,H2_* coupling constants (2.5 Hz) and *J_C1,H1_* coupling constant (170Hz) [14,15]. The L-rhamnose was determined to have the α-conﬁguration based on the broad singlet of its anomeric proton [14,15]. The sugar linkage at C-3 were determined on the basis of the HMBC spectrum which showed correlation between a proton signal at δ 5.27 (glc-H-1) and a carbon signal at δ 85.1 due to C-3 of the aglycone moiety (see Figure 2). The sequence of the sugar chain at C-28 was established from the following HMBC correlations between H-1 (δ 5.18) of terminal xylose and C-4 (δ 83.6) of rhamnose, H-1 (δ 5.78) of rhamnose and C-2 (δ 75.2) of arabinose, H-1 (δ 6.47) of arabinose and C-28 (δ 175.9) (Figure 2). On the basis of the above evidence, platycoside O (1) was identiﬁed to be 3-*O*-β-D-glucopyranosyl-2β,3β,16α,23-tetrahydroxyolean-12-en-24-methoxyl, 24-oxo-28-oic acid 28-*O*-β-D-xylopyranosyl-(1→4)-α-L-rhamnopyranosyl-(1→2)-α-L-arabinopyranoside.

The four known saponins were identified as platycoside M-3 (2), platycoside J (3), platycoside F (4), and platycoside B (5) through comparison of their UV, IR, NMR and MS data with literature values [14,15].

**Table 1:** ^13^C and ^1^H -NMR data of **1** in pyridine-*d_5_* (500 MHz for H, 150 MHz for C).

No	C	H			No	C	H
1	45.1	1.45 (1H, o),			3–O–Glu		
		2.03–2.12 (1H, m)			1	106.4	5.27 (1H, d, *J* = 8.0 Hz)
2	70.2	4.77 (1H, br s)			2	74.9	3.99 (1H, t, *J* = 9.0 Hz)
3	85.1	4.58 (1H, br s)			3	78.6	4.05 (1H, t, *J* = 7.5, 9.0 Hz)
4	48.2				4	73.0	4.37–4.42 (1H, m)
5	47.8	1.90–1.94 (1H, m)			5	77.0	4.46–4.49 (1H, m)
6	19.1	1.92–1.94 (1H, m);			6	63.2	4.83 (1H, br d, *J* = 11.0 Hz)
		1.30–1.35 (1H, m)					3.95 (1H, dd, *J* = 4.5, 11.0 Hz)
7	33.7	1.65–1.76(1H, m);					
		1.48 (1H, d–like, *J* = 11.5 Hz)			C–28–Ara		
8	40.3				1	93.6	6.47 (1H, d, *J* = 2.5 Hz)
9	47.8	1.90–1.94 (1H, m)			2	75.2	4.53–4.58 (1H, m)
10	37.2				3	70.1	4.50–4.54 (1H, m)
11	24.2	2.08 (1H, d–like, *J* = 17.0 Hz);			4	66.2	4.37–4.45 (1H, m)
		2.14 (1H, d–like, *J* = 17.5 Hz)			5	63.2	4.53–4.56 (1H, m)
12	123.1	5.63 (1H, t–like)					4.26 (1H, br d, *J* = 13.5 Hz)
13	144.4						
14	42.3				Rha		
15	36.1	2.31 (1H, d–like, *J* = 12.0 Hz)			1	101.2	5.78 (1H, br s)
		1.81 (1H, dd, *J* = 3.0, 15.0 Hz)			2	72.0	4.57 (1H, br d, *J* = 3.0 Hz)
16	74	5.25 (1H, d–like, *J* = 3.6 Hz)			3	72.7	4.61 (1H, dd, *J* = 3.0, 8.5 Hz)
17	49.6				4	83.6	4.39 (1H, t, *J* = 9.5 Hz)
18	41.4	3.57 (1H, dd, *J* = 4.0, 14.0 Hz)			5	68.6	4.42 (1H, dq, *J* = 5.5, 9.5 Hz)
19	47.1	2.75 (1H, t–like, *J* = 13.0, 14.0 Hz)			6	18.4	1.74 (3H, d, *J* = 5.5 Hz)
		1.35 (1H, dd, *J* = 4.0, 13.0 Hz)					
20	30.9				Xyl		
21	36	2.40 (1H, dt, *J* = 5.0, 12.0 Hz)			1	106.8	5.18(1H, d, *J* = 8.0 Hz)
		1.29 (1H, d–like, *J* = 11.5 Hz)			2	76.0	4.03–4.08 (1H, m)
22	32.1	2.25–2.35 (1H, m);			3	77.8	4.18 (1H, t, *J* = 8.5 Hz)
		2.20 (1H, dd, *J* = 5.5, 12.5 Hz)			4	71.0	4.07–4.24 (1H, m)
23	63.7	5.02 (1H, o),4.09 (1H, m)			5	67.4	4.15–4.25 (1H, m)3.50 (1H, t–like, *J* = 11.0 Hz)
24	170.5						
25	17.8	1.55 (3H, s)					
26	17.6	1.14 (3H, s)					
27	27.2	1.76 (3H, s)					
28	175.9						
29	33.3	0.99 (3H, s)					
30	24.8	1.14 (3H, s)					
–OCH_3_	52.1	3.69 (3H, s)					

## 3. Experimental

### 3.1. General

FAB-MS and HR-FAB-MS spectral were recorded on a JEOL JMS-SX 102A mass spectrometer. IR spectra were measured with a Bruker IFS-55 infrared spectrometer. ^1^H- and ^13^C-NMR spectra were recorded with a JEOL α 500/600 FT NMR spectrometer. Chemical shifts were reported in parts per million on the d scale with TMS as an internal standard. Silica gel (Qingdao Haiyang Chemical Co., Ltd. 200–300 mesh) and Lichroprep RP-18 (Merck) were used for silica gel column chromatography and MPLC. Preparative HPLC was performed using an octadecyl silica (ODS) column (Pegasil ODS, Senshu Pak, 250 mm × 10 mm i.d.) on a Hitachi liquid chromatography system with an RI detector. Gas liquid chromatography was carried out on a Shimadzu GC-7A under the following conditions: column, 3% ECNSS-M (2 m × 0.3 mm); column temperature, 190 °C; injection temperature, 210 °C; carrier gas, N_2_; and ﬂow rate, 25 mL/min. Spots were visualized by spraying with ethanol–10% H_2_SO_4_ and heating (110 °C, 5 min).

### 3.2. Plant Material

The roots of *P. grandiﬂorum* were collected from Shenyang, Liaoning Province, China, in 2003 and were taxonomically identiﬁed by Professor Sun Qi-Shi of Shenyang Pharmaceutical University. A voucher specimen (No. 20030321) is deposited at the Herbarium of Shenyang Pharmaceutical University.

### 3.3. Extraction and Isolation

The air-dried roots of *P. grandiﬂorum* (10 kg) were pulverized and extracted with 75% EtOH (ethanol:water = 3:1, v/v) three times under reﬂux. The combined extract was evaporated *in vacuo*, suspended in water, and then partitioned with EtOAc. The aqueous layer was chromatographed over a macroporous resin D101 column and eluted with H_2_O, 60% EtOH, and 95% EtOH. The 60% EtOH elution was evaporated under a vacuum to obtain a residue (180 g). The residue (80 g) was fractioned on silica gel (solvent, CHCl_3_-MeOH = 50:1–1:1, v/v) to give four fractions (Fr.I-IV). Fraction III (27.0 g) was applied onto a silica gel column (solvent, CHCl_3_-MeOH-H_2_O = 30:10:1 → 6:4:1, v/v/v) to give a crude saponin sub-fractions (Fr A-C). Fraction C (18.0 g) was chromatographed on a silica gel column (solvent, EtOAc- EtOH-H_2_O = 9:1:0.5 → 85:15:7.5, v/v/v), followed by MPLC [Lichroprep RP-18 (Merck), solvent, MeOH-H_2_O (2:3 → 7:3, v/v)] and ﬁnally by semi-preparative HPLC (MeOH-H_2_O = 52:48) to give three new compounds **1** (7.6 mg), **2** (8.1 mg) and **3** (8.2 mg). Further puriﬁcation of Fraction B (5.0 g) by MPLC [Lichroprep RP-18 (Merck), solvent, MeOH-H_2_O (2:3 → 7:3, v/v)] and ﬁnally by semi-preparative HPLC (MeOH-H_2_O = 45:55, v/v) to give three compounds **4 **(15 mg) and **5** (6.2 mg). 

### 3.4. Characterization of Platycoside O (1)

Obtained as a white amorphous powder; IR ν_max_: 3421, 2926, 1738, 1633, 1384, 1226 and 1042 cm^−1^; HR-FAB-MS *m/z*: 1143.5130 [M+Na]^+^ (Calcd. for C_53_H_84_O_25_Na, 1143.5200); ^1^H-NMR (pyridine-*d_5_*, 500 MHz) and ^13^C-NMR (pyridine-*d_5_*, 150 MHz): see Table 1. 

### 3.5. Acid Hydrolysis of 1

Compound **1** (2.0 mg) were heated 1M HCl (dioxane–H_2_O, 1:1, 1 mL) at 90 °C for 3 h in a water bath. Dioxane was removed, the solution was extracted with EtOAc (1 mL × 3), and the EtOAc was removed. The monosaccharide portions were analyzed by gas chromatography after conversion of the hydrolysates into corresponding alditol acetates. The arabinitol, glucitol, rhamnitol and xylitol acetates from compound **1** were detected in a ratio of 1:1:1:1 respectively using gas chromatography analysis. The absolute conﬁgurations of the sugars were determined according to the method reported by Hara and coworkers [14,15] using gas chromatography with the following conditions: column: 3% ECNSS-M (2 m × 0.3 mm); column temperature: 190 °C; injection temperature: 210 °C. The absolute conﬁgurations of the sugars were determined as D-xylose, L-arabinose, L-rhamnose and D-glucose by comparison of its retention times with those of authentic sample [14,15]. 

## 4. Conclusions

During the phytochemical survey of the roots extract of *Platycodon grandiflorum*, a new unusual minor triterpenoid saponin **1** was isolated and identified as platycoside O on the basis of spectral analysis and chemical evidence.
